# Supplementary material for: Electron cascade for distant spin readout
Source: Nat Commun. 2021 Jan 4;12:77. doi: 10.1038/s41467-020-20388-6 (PMC7782677; doi:10.1038/s41467-020-20388-6)
Supplement: Supplementary file 1 — Supplementary Information [file 41467_2020_20388_MOESM1_ESM.pdf]

Supplementary Information for
Electron cascade for distant spin readout

Cornelis J. van Diepen[1], Tzu-Kan Hsiao[1], Uditendy Mukhopadhyay[1], Christian Reichl[2], Werner Wegscheider[2], Lieven M. K. Vandersypen[1]

[1]QuTech and Kavli Institute of Nanoscience, Delft University of Technology, 2600 GA Delft, The Netherlands
[2]Solid State Physics Laboratory, ETH Zürich, 8093 Zürich, Switzerland

TABLE OF CONTENTS

Supplementary Note 1. SIGNAL IN CASCADE CSD 2

Supplementary Note 2. OPERATING WINDOW FOR CASCADE READOUT 2
A. Pauli spin blockade 2
B. Cascade with dot-reservoir 2
C. Cascade with inter-dot 2
D. At higher filling 3

Supplementary Note 3. SINGLE-SHOT HISTOGRAM 3

Supplementary Note 4. INTER-DOT CASCADE PAULI SPIN BLOCKADE 3

Supplementary Note 5. THEORY ON CASCADE SPEED AND SUCCESS PROBABILITY 3
A. Co-tunnel cascade Pauli spin blockade 4
B. Co-tunnel inter-dot cascade Pauli spin blockade 5
C. Controlled propagation 5
D. Longer cascade 5
E. Scaling of success probability 6

Supplementary Note 6. RELAXATION AND EXCITATION TIME 7

Supplementary Note 7. FIDELITY ANALYSIS FOR PSB 7

Supplementary Note 8. EFFECT OF HYPERFINE FIELD 7

Supplementary Note 9. SPIN FUNNEL 7

References 7

Supplementary Note 1. SIGNAL IN CASCADE CSD

The signal for Fig. 2b in the main text was taken with the fourth dot and the sensor tuned such that all charge occupations result in clearly distinguishable signals. The signal for (1101) is higher than that for (0101), because the rightmost dot was close to the Fermi level for (1101), and thus only partially occupied. The signal for (1200) is higher than for (0200), because the signal for these occupations is from the high-voltage flank of a sensing dot Coulomb peak. The other relative signals are as intuitively expected, namely adding charges and bringing charges closer to the sensor both result in a reduced sensor signal.

Supplementary Note 2. OPERATING WINDOW FOR CASCADE READOUT

In order to get insight in the size of the operating window for PSB, CPSB and inter-dot CPSB (iCPSB), we start from the single-band Fermi-Hubbard Hamiltonian for the quantum dot array [1]:

$$H = -\sum_i \epsilon_i n_i + \sum_i \frac{U_i}{2} n_i(n_i - 1) + \sum_{ij, i\neq j} V_{ij} n_i n_j - \sum_{\langle i,j \rangle} t_{c,ij} \left(c_i^\dagger c_j + h.c. \right), \tag{1}$$

where ϵ_i is the single-particle energy offset, $n_i = c_i^\dagger c_i$ is the dot occupation, and $c_i^{(\dagger)}$ is the annihilation (creation) operator, U_i is the on-site Coulomb repulsion, and V_{ij} the inter-site Coulomb repulsion. For simplicity, we assume in what follows homogeneous Coulomb repulsion, thus $U_i = U$, $V_{i,i+1} = V$, $V_{i,i+2} = V'$, and $V_{i,i+3} = V''$, and neglect tunnel coupling. The shifts of charge transition lines due to capacitive couplings, and the tuning of dot potentials for each of the different readout schemes, namely PSB, CPSB and iCPSB, are obtained by solving sets of constraints. Note that, because the interaction strength decays with distance, the size of the operating window for a charge transition only depends on the occupation of nearby dots. This implies that the sizes of the operating windows remain constant as the length of the cascade is extended.

A. Pauli spin blockade

The on-site potential ϵ_1 must satisfy $\mu_1(1200) > 0 > \mu_1(1100)$, which yields $2V > \epsilon_1 > V$. The constraint for ϵ_2 follows from $E(1200) > E(1100) > E(0200)$, which yields $U + V > \epsilon_2 > \epsilon_1 + U - V$. From these constraints it follows that the shifts of the relevant charge transition lines due to capacitive couplings are V when projected onto ϵ_1 or ϵ_2. For the two dots on the right to remain empty $\mu_3(1010) > 0$ and $\mu_4(1001) > 0$, which respectively yield $\epsilon_3 < V'$ and $\epsilon_4 < V''$.

B. Cascade with dot-reservoir

The constraint for dot 1 is now $\mu_1(1200) > 0 > \mu_1(1101)$, which yields $2V > \epsilon_1 > V + V''$. We also require $E(1200) > E(1101) > E(0200)$, which yields $\epsilon_4 + U + V - V' - V'' > \epsilon_2 > \epsilon_1 + \epsilon_4 + U - V - V' - V''$. For the rightmost dot, the cascade occurs when $\mu_4(0201) > 0 > \mu_4(1101)$, which yields $2V' > \epsilon_4 > V' + V''$. From these constraints it follows that the shifts of the relevant charge transition lines are $V - V''$ when projected onto ϵ_1 or ϵ_2, and $V' - V''$ when projected onto ϵ_4 or onto $\epsilon_1 - \epsilon_2$. For the third dot to remain empty $\mu_3(1110) > 0$, which yields $\epsilon_3 < V'$.

C. Cascade with inter-dot

For the leftmost dot $\mu_1(1201) > 0 > \mu_1(1110)$, which yields $2V + V'' > \epsilon_1 > V + V'$. Another requirement is $E(1201) > E(1110) > E(0201)$, which yields $\epsilon_3 - \epsilon_4 + U + V'' > \epsilon_2 > \epsilon_1 + \epsilon_3 - \epsilon_4 + U - 2V + V'$. For the two dots on the right, the cascade effect takes place when $\mu_3(1110) < \mu_4(1101)$, which yields $\epsilon_3 - \epsilon_4 > V - V''$, and $\mu_3(0210) > \mu_4(0201)$, which yields $\epsilon_3 - \epsilon_4 < 2V - 2V'$. In addition, for the rightmost dot, we need $\mu_4(0201) < 0 < \mu_4(1111)$, which yields $2V' < \epsilon_4 < V + V' + V''$. From these constraints it follows that the shifts of the relevant charge transition lines are $V - V' + V''$ when projected onto ϵ_1 or ϵ_2, and $V - 2V' + V''$ when projected onto $\epsilon_1 - \epsilon_2$ or $\epsilon_3 - \epsilon_4$.

D. At higher filling

For the cascade-based readout in an array with all sites initially occupied the charge occupation after the cascade is $(1\ldots1)$ or $(21\ldots10)$. The electron-electron interaction decays with distance, thus may be too weak to prevent the $(1\ldots1)$ state from becoming a $(21\ldots1)$ state, while $(21\ldots10)$ is preserved, or similarly to prevent $(21\ldots10)$ from becoming $(21\ldots1)$, while $(1\ldots1)$ is preserved. The unwanted loading of electrons can be suppressed by operating the cascade with low couplings to reservoirs or in the isolated regime [2, 3].

Supplementary Note 3. SINGLE-SHOT HISTOGRAM

The single-shot histograms are modeled with [4]

$$N(x) = N_{\text{tot}}\left[P_S n_S(x) + (1-P_S)n_T(x)\right] w_{\text{bin}} \tag{2}$$

with N_{tot} the total number of single-shot repetitions, P_S the average singlet probability over al single-shot outcomes, w_{bin} the bin width, and n_S and n_T the probability density distribution for the singlet and triplet states respectively. The probability density distributions are modeled by noise-broadened Gaussians as

$$n_S(x) = \frac{1}{\sqrt{2\pi}\sigma} e^{-(x-\mu_S)^2/2\sigma^2}, \tag{3}$$

and

$$n_T(x) = \frac{1}{\sqrt{2\pi}\sigma}\left[e^{-t_{\text{int}}/T_1} e^{-(x-\mu_T)^2/2\sigma^2} + \frac{t_{\text{int}}}{T_1}\int_{\mu_T}^{\mu_S} \frac{1}{\mu_S-\mu_T} e^{-\left[(x'-\mu_T)/(\mu_S-\mu_T)\right](t_{\text{int}}/T_1)} e^{-(x-x')^2/2\sigma^2} dx'\right], \tag{4}$$

where the second term accounts for relaxation during the integration time, where μ_S and μ_T are the means and σ the standard deviation of the gaussians for the singlet and triplet density distributions respectively. T_1 is the triplet-singlet relaxation time obtained from an exponential fit to the averaged data as shown in the insets of Fig. 3, and t_{int} is the integration time.

From the probability density distributions we obtain the uncorrected readout fidelities as

$$F_S = 1 - \int_{V_T}^{\infty} n_S(x)dx, \qquad F_T = 1 - \int_{-\infty}^{V_T} n_T(x)dx, \tag{5}$$

with V_T the signal threshold, and $F_{\text{avg}} = \frac{1}{2}(F_S + F_T)$. These uncorrected readout fidelities include errors due to residual overlap of the histograms and relaxation during the integration time. We afterwards correct the readout fidelity for errors due to relaxation during the arming time, and errors due to excitation during the integration and arming time as described in the main text.

Supplementary Note 4. INTER-DOT CASCADE PAULI SPIN BLOCKADE

An alternative implementation for cascade-based readout in a quadruple dot is shown in Supplementary Fig. 1. The additional electron moves from the third dot to the fourth dot, thus the cascade involves an inter-dot transition. The signal is from the left flank of a Coulomb peak of the sensing dot. The signal changes of the two charge transitions now add up, thus a singlet state corresponds, as with Pauli spin blockade, to the peak at lower signal and a triplet state corresponds to the peak at higher signal.

Supplementary Note 5. THEORY ON CASCADE SPEED AND SUCCESS PROBABILITY

In order to assess the scalability of the cascade-based readout, we analyse the speed and adiabaticity of the movement of charges in the cascade. The speed of the cascade is important since spin measurement must be faster than spin relaxation for achieving high-fidelity spin readout. Furthermore, spin readout must be faster than spin decoherence (with dynamical decoupling) for achieving fault-tolerance using feedback in quantum error correction. The adiabaticity with respect to charge is important when the Zeeman splitting is different between quantum dots. For different Zeeman splitting, the uncertainty in the electron position results in a phase error.

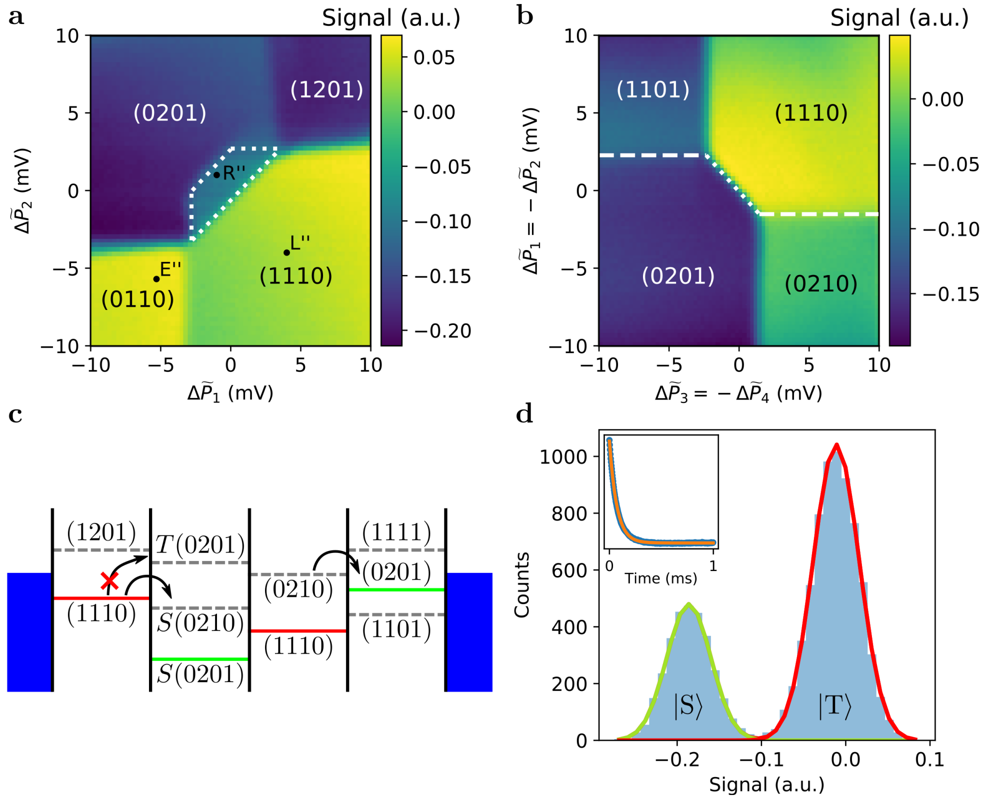

Supplementary Fig. 1. **Cascade-based readout with inter-dot transition.** Numbers in round brackets indicate charge occupations of the dots. **a** Charge-stability diagram as a function of the virtual plunger gates of dots 1 and 2. The trapezoid on the top-left side of the inter-dot transition is the inter-dot cascade Pauli spin blockade (iCPSB) window. The black dots indicate the voltages for the iCPSB readout cycle: E$''$(mpty), L$''$(oad) and R$''$(ead). **b** Charge-stability diagram showing the effect of the inter-dot transition for dots 1 and 2 on the inter-dot transition for dots 3 and 4. On the left and the right, the electron in dot 3 or 4 stays in place when an electron moves from dot 1 to dot 2. In the center, an electron moves from dot 3 to dot 4 when the electron on dot 1 is pushed to dot 2. This corresponds to an inter-dot cascade effect. **c** Ladder diagram corresponding to the readout point R$''$, illustrating the tuning of the dot potentials for the cascade Pauli spin blockade with inter-dot transition. Note that $\mu_{2,S}(0210)$ is drawn below $\mu_1(1110)$, but for the cascade it could also be above. **d** Histograms and fits of 10,000 single-shot measurements for iCPSB readout. The integration time is $t_{int} = 1.5\,\mu\text{s}$. Red and green solid lines correspond to the respectively triplet and singlet probability distributions, obtained from the fit to the histogram [4, 5]. For iCPSB readout the singlet corresponds to charge occupation (0201) and the triplet to (1110). The inset shows the signal averaged over the single-shots and an exponential fit, with $T_1 = (75.0 \pm 0.2)\,\mu\text{s}$.

A. Co-tunnel cascade Pauli spin blockade

When the cascade is operated such that $E(S(0201)), E(1100) > E(1101)$, then the cascade occurs via a co-tunnel process, and the cascade can be operated adiabatically. For a quantum dot array with length four and when the cascade involves a dot-reservoir transition, the relevant charge states are (1101), (0200), (1100) and (0201). The Hamiltonian in this basis is

$$\begin{pmatrix} -\epsilon_1-\epsilon_2-\epsilon_4+V+V'+V'' & 0 & -t_{c,4R} & -\widetilde{t}_{c,12} \\ 0 & -2\epsilon_2+U & -\widetilde{t}_{c,12} & -t_{c,4R} \\ -t_{c,4R} & -\widetilde{t}_{c,12} & -\epsilon_1-\epsilon_2+V & 0 \\ -\widetilde{t}_{c,12} & -t_{c,4R} & 0 & -2\epsilon_2-\epsilon_4+U+2V' \end{pmatrix}, \tag{6}$$

with $\widetilde{t}_{c,12} = \sqrt{2}t_{c,12}$, and $t_{c,4R}$ the tunnel coupling between the rightmost dot and the right reservoir. Rewrite the Hamiltonian as

$$\begin{pmatrix} \frac{\widetilde{\epsilon}}{2} & 0 & -t_{c,4R} & -\widetilde{t}_{c,12} \\ 0 & -\frac{\widetilde{\epsilon}}{2} & -\widetilde{t}_{c,12} & -t_{c,4R} \\ -t_{c,4R} & -\widetilde{t}_{c,12} & \frac{V'-V''+\widetilde{\delta}}{2} & 0 \\ -\widetilde{t}_{c,12} & -t_{c,4R} & 0 & \frac{V'-V''-\widetilde{\delta}}{2} \end{pmatrix}, \tag{7}$$

with $\widetilde{\epsilon} = \epsilon - U + V + V' + V''$, and $\widetilde{\delta} = \delta - U + V - 2V'$, where $\epsilon = \epsilon_{12} + \epsilon_4$, and $\delta = \epsilon_{12} - \epsilon_4$, with $\epsilon_{12} = -\epsilon_1 + \epsilon_2$. By diagonalising this Hamiltonian, with approximation $|\widetilde{\epsilon}| \ll |V' - V'' \pm \widetilde{\delta}|$, the co-tunnel coupling between the eigenstates that are predominantly (1101) and (0200) is $t_{co} = \frac{\widetilde{t}_{c,12}t_{c,4R}}{\Delta_+} + \frac{\widetilde{t}_{c,12}t_{c,4R}}{\Delta_-}$, with $\Delta_\pm = \frac{V'-V''\pm\widetilde{\delta}}{2}$ [6].

B. Co-tunnel inter-dot cascade Pauli spin blockade

The analysis for a cascade involving co-tunnelling and only inter-dot transitions is very similar as for the co-tunnel cascade with a dot-reservoir transition. For cascade with an inter-dot transition, the relevant charge states are (1110), (0201), (1101) and (0210). The Hamiltonian in this basis is

$$\begin{pmatrix} -\epsilon_1 - \epsilon_2 - \epsilon_3 + 2V + V' & 0 & -t_{c,34} & -\widetilde{t}_{c,12} \\ 0 & -2\epsilon_2 - \epsilon_4 + U + 2V' & -\widetilde{t}_{c,12} & -t_{c,34} \\ -t_{c,34} & -\widetilde{t}_{c,12} & -\epsilon_1 - \epsilon_2 - \epsilon_4 + V + V' + V'' & 0 \\ -\widetilde{t}_{c,12} & -t_{c,34} & 0 & -2\epsilon_2 - \epsilon_3 + U + 2V \end{pmatrix}. \tag{8}$$

Rewrite the Hamiltonian as

$$\begin{pmatrix} \frac{\widetilde{\epsilon}}{2} & 0 & -t_{c,34} & -\widetilde{t}_{c,12} \\ 0 & -\frac{\widetilde{\epsilon}}{2} & -\widetilde{t}_{c,12} & -t_{c,34} \\ -t_{c,34} & -\widetilde{t}_{c,12} & \frac{V-2V'+V''+\widetilde{\delta}}{2} & 0 \\ -\widetilde{t}_{c,12} & -t_{c,34} & 0 & \frac{V-2V'+V''-\widetilde{\delta}}{2} \end{pmatrix}. \tag{9}$$

with $\widetilde{\epsilon} = \epsilon - U + 2V - V'$ and $\widetilde{\delta} = \delta - U - V + V' + V''$, where $\epsilon = \epsilon_{12} + \epsilon_{34}$, and $\delta = \epsilon_{12} - \epsilon_{34}$ with $\epsilon_{ij} = -\epsilon_i + \epsilon_j$. By diagonalising this Hamiltonian, with approximation $|\widetilde{\epsilon}| \ll |V - 2V' + V'' \pm \widetilde{\delta}|$, the co-tunnel coupling between the eigenstates that are predominantly (1110) and (0201) is $t_{co} = \frac{\widetilde{t}_{c,12}t_{c,34}}{\Delta_+} + \frac{\widetilde{t}_{c,12}t_{c,34}}{\Delta_-}$, with $\Delta_\pm = \frac{V-2V'+V''\pm\widetilde{\delta}}{2}$.

C. Controlled propagation

The cascade can be implemented such that the propagation is controlled by a sequence of gate voltages. As example, we consider the cascade Pauli spin blockade as described in the main text. First, conventional PSB is performed with $\mu_4(0201) < 0$. The electron on the fourth dot remains there. Next, gate voltages are changed such that $\mu_4(1101) < 0 < \mu_4(0201)$. Then the cascade will propagate and the electron on the fourth dot will move to the reservoir. A similar scheme can be designed for inter-dot cascade PSB. For a longer cascade path, which involves more than two charge transitions, the propagation could be controlled at each transition. The motivation for controlled propagation becomes clear in the next subsection.

D. Longer cascade

We now discuss how the total duration of the cascade scales with the length of the cascade path for three different scenarios.

First we consider a cascade where all the charges are displaced in one single co-tunnel process. This involves N simultaneous tunnel events that are each energetically forbidden, but where the final state is lower in energy than the initial state. Then, for a chain with length $2N$ (with every other site occupied, except for the first two sites where the PSB mechanism is implemented), and homogeneous tunnel coupling, $t_{c,ij} = t_c$, and when the cascade involves only inter-dot transitions between neighbouring pairs, the co-tunnel coupling is [7]

$$t_{co} = N! \frac{\sqrt{2} t_c^N}{2V^{N-1}}, \tag{10}$$

where for simplicity we only included inter-site Coulomb repulsion between nearest-neighbour sites. Charge adiabaticity will require increasingly slower gate voltage changes, because $t_c < V$, thus t_{co} decreases exponentially with increasing cascade length. When the adiabaticity condition is not met, the cascade can get stuck along the way.

Next, for the sequential tunneling regime, thus with $E(11LL\dots L) > E(02LL\dots L) > E(02RL\dots L) > \dots > E(02RR\dots R)$, with $L = 10$ and $R = 01$, the expected duration, assuming homogeneous tunnel rates, Γ, for the individual transitions is [8]

$$\langle \tau \rangle \sim \frac{N}{\Gamma}. \tag{11}$$

For the sequential regime, charge adiabaticity need not be preserved. Charge tunnelling is here a stochastic process and the duration only scales linearly with the length. Note that there is an intermediate regime, which does not fully rely on co-tunnelling, but is also not completely sequential. Theory on this regime is beyond the scope of this work.

Finally, both the charge adiabaticity and speed can be largely maintained in a cascade with controlled propagation. The total duration of the cascade increases linearly with the cascade length, similar to the sequential case, but now uncertainties in the timing of the charge movement can be suppressed, which is important when the Zeeman splittings are not homogeneous along the path.

Alternatively, co-tunnel, sequential, and cascades with controlled propagation could be combined, such that different parts of the cascade have different character.

E. Scaling of success probability

Here we consider the probability density function for the total cascade duration and its scaling with cascade length in the context of charge getting stuck. The probability density function for the cascade duration with $N+1$ transitions, which each have decay rate Γ, is given by the Erlang distribution [9]

$$P_N(t) = \frac{\Gamma^{N+1} t^N}{N!} e^{-\Gamma t}. \tag{12}$$

The probability for the cascade to take longer than time t is

$$\int_t^\infty P_N(t') \mathrm{d}t' = e^{-\tau} \sum_{n=0}^{N} \frac{\tau^n}{n!}, \tag{13}$$

with $\tau = \Gamma t$. We will show that the probability for a cascade with $N+1$ transitions to take longer than time τ_N decreases for increasing N given $\tau_N > \frac{N}{N-1} \tau_{N-1}$, with τ_i the time used to obtain the probability for the cascade of length $i+1$. From the constraint on τ_N it follows that $\tau_N \geq N\tau_1$, thus we consider $\tau_N = N\tau_1$, because if the scaling holds for this τ_N, then it will certainly hold for $\tau_N > N\tau_1$. The derivative of the probability with respect to N is

$$\frac{\mathrm{d}}{\mathrm{dN}} \left[e^{-\tau_N} \sum_{n=0}^{N} \frac{\tau_N^n}{n!} \right] = \frac{\mathrm{d}}{\mathrm{dN}} \left[e^{-N\tau_1} \sum_{n=0}^{N} \frac{N^n \tau_1^n}{n!} \right] = -\tau_1 e^{-N\tau_1} \frac{N^N \tau_1^N}{N!}. \tag{14}$$

Thus the probability decreases as function of cascade length, which shows that the time for a cascade to complete with a given probability scales sublinearly with respect to the cascade length. Alternatively formulated, the probability for a charge to get stuck and interrupt the cascade thus scales sublinearly with the cascade length.

Supplementary Note 6. RELAXATION AND EXCITATION TIME

The relaxation and excitation time are obtained from the signal averaged over the single-shot measurements, and as a function of time. This signal is fitted with an exponential [10]

$$V(t) = A\exp(-\Gamma t) + B, \tag{15}$$

with A a pre-factor,

$$\Gamma = \frac{T_1 + T_{exc}}{T_1 T_{exc}}, \tag{16}$$

and

$$B = \frac{1}{\Gamma}\left(\frac{V_T}{T_{exc}} + \frac{V_S}{T_1}\right), \tag{17}$$

where V_T and V_S are obtained from the fit to the histogram of the singlet-shot measurements.

Supplementary Note 7. FIDELITY ANALYSIS FOR PSB

From the fit to the histogram in Fig. 3a, the error due to overlap and relaxation during integration is $\eta_{hist} = 14.3\%$. The relaxation time is $T_1 = (724 \pm 70)\,\mu\text{s}$, which results in an error of $\eta_{arm} = 0.014\%$. The excitation time is $T_{exc} = (2.8 \pm 1.1)\,\text{ms}$, which results in an error of $\eta_{exc} = 0.030\%$. The error due to charge non-adiabaticity is the same as for CPSB, thus $10^{-9}\%$.

Supplementary Note 8. EFFECT OF HYPERFINE FIELD

The measurement basis for spin readout consists of the singlet and triplet states, which are the eigenstates of the Hamiltonian at the readout point. The voltage pulse from the loading point to the readout point, induces a mapping of the eigenstates at the loading point to the measurement basis. This mapping is determined by the pulse duration, the exchange coupling, and the hyperfine field, which is caused by the hyperfine interaction of the electron spins with the nuclear spins. The eigenstates at the loading point can vary between pulse cycles, due to fluctuations of the hyperfine field, thus changing the mapping to the measurement basis. In order to avoid unpredictable mappings, the exchange interaction must dominate the Hamiltonian. This can be done by increasing the exchange interaction or suppressing the hyperfine field fluctuations, either by feedback mechanisms based on dynamical nuclear polarization [11] or by using isotopically purified ^{28}Si[12].

Supplementary Note 9. SPIN FUNNEL

The strength of the tunnel coupling between dots 1 and 2, $t_{c,12}$, is obtained from a so-called spin funnel measurement, which is shown in Supplementary Fig. 2. For the spin funnel, a pulse cycle with three stages is executed [13]. The first stage is deep in the (0200) charge region to initialise a singlet state. Then the voltages are pulsed towards the (1100) region, and then into the readout region in (0200). Such a pulse cycle is repeated for varying depths in the (1100) region and varying external magnetic fields. The magnetic field is converted to an energy scale with the g-factor, $|g| = 0.44$, and the Bohr magneton. From a fit to the funnel, the tunnel coupling $t_{c,12} = 11.5\,\mu\text{eV}$ is obtained. The detuning is obtained from the change in virtual gate voltages by multiplying with the lever arms, which were obtained with photon-assisted tunnelling measurements [14].

[1] Hensgens, T. *et al.* Quantum simulation of a Fermi-Hubbard model using a semiconductor quantum dot array. *Nature* **548**, 70–73 (2017).

[2] Bertrand, B. *et al.* Quantum manipulation of two-electron spin states in isolated double quantum dots. *Physical Review Letters* **115**, 096801 (2015).

[3] Yang, C. H. *et al.* Operation of a silicon quantum processor unit cell above one kelvin. *Nature* **580**, 350-354 (2020).

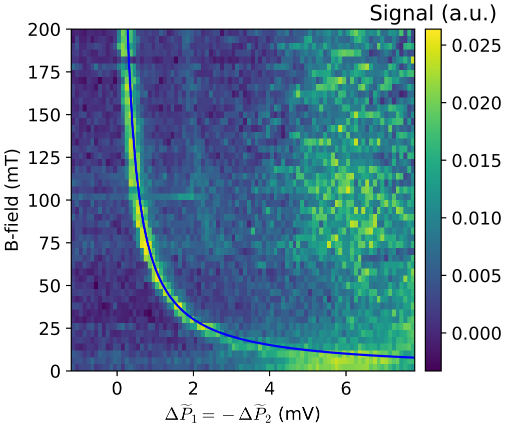

Supplementary Fig. 2. **Spin funnel** Higher signal corresponds to a higher triplet probability. For each data point the signal is averaged over 1,000 single-shot measurements. The wait time at the operating point, which is in the (1100) charge configuration for $\Delta\widetilde{P}_1 > 0$, was 200 ns. The blue, solid line is a fit to $\frac{1}{2}\left(-\epsilon_{12} + \sqrt{8t_{c,12}^2 + \epsilon_{12}^2}\right)$, with $\epsilon_{12} = -\epsilon_1 + \epsilon_2$, the detuning, and $t_{c,12}$ the tunnel coupling between dots 1 and 2.

[4] Barthel, C., Reilly, D. J., Marcus, C. M., Hanson, M. P. & Gossard, A. C. Rapid single-shot measurement of a singlet-triplet qubit. *Physical Review Letters* **103**, 1–4 (2009).
[5] Zheng, G. *et al.* Rapid gate-based spin read-out in silicon using an on-chip resonator. *Nature Nanotechnology* **14**, 742–746 (2019).
[6] Braakman, F. R., Barthelemy, P., Reichl, C., Wegscheider, W. & Vandersypen, L. M. K. Long-distance coherent coupling in a quantum dot array. *Nature Nanotechnology* **8**, 432–437 (2013).
[7] Averin, D. V. & Odintsov, A. A. Macroscopic quantum tunneling of the electric charge in small tunnel junctions. *Physics Letters A* **140**, 251–257 (1989).
[8] Buttiker, M. Coherent and Sequential Tunneling in Series Barriers. *IBM Journal of Research and Development* **32**, 63–75 (1988).
[9] D'Anjou, B. & Coish, W. A. Enhancing qubit readout through sub-Poissonian dynamics. *Physical Review A* **96**, 052321 (2017).
[10] Harvey-Collard, P. *et al.* High-Fidelity Single-Shot Readout for a Spin Qubit via an Enhanced Latching Mechanism. *Physical Review X* **8**, 2–5 (2018).
[11] Bluhm, H., Foletta, S., Mahalu, D., Umansky, V. & Yacoby, A. Enhancing the Coherence of a Spin Qubit by Operating it as a Feedback Loop Controlling its Nuclear Spin Bath. *Physical Review Letters* **105**, 216803 (2010).
[12] Veldhorst, M. *et al.* An addressable quantum dot qubit with fault-tolerant control-fidelity. *Nature Nanotechnology* **9**, 981-985 (2014).
[13] Petta, J. R. *et al.* Coherent Manipulation of Coupled Electron Spins in Semiconductor Quantum Dots. *Science* **309**, 2180–2184 (2005).
[14] Hsiao, T.- K. *et al.* Efficient orthogonal control of tunnel couplings in a quantum dot array. *Physical Review Applied* **13**, 054018 (2020).
